# CRISPR Genome Editing: How to Make a Fantastic Method Even Better

**DOI:** 10.3390/cells10020408

**Published:** 2021-02-16

**Authors:** Cord Brakebusch

**Affiliations:** Biotech Research and Innovation Center (BRIC), University of Copenhagen, Ole Maaløes Vej 5, 2200 Copenhagen, Denmark; cord.brakebusch@bric.ku.dk

CRISPR genome editing describes targeted mutagenesis involving a programmable DNA scissor consisting of a protein (Cas9) bound to a short RNA. The RNA, called guide RNA (gRNA), determines the target site in the genome by complementary base paring, while the Cas9 protein is required for the induction of a double-strand DNA break (DSB) at the binding site. In the absence of a DNA template, the DSB is repaired in an error-prone manner, which is used to induce gene knockouts and deletions. In the presence of a DNA template with homologous arms, i.e., DNA sequences identical to those flanking the targeting site, foreign DNA can be introduced to the region close to the DSB by homologous recombination. This repair is error-free and can be used, for example, for the insertion of point mutations or reporter genes.

Due to its simplicity, CRISPR quickly became a standard technique in biomedical research. The high efficiency of CRISPR genome editing has further raised high hopes for the generation of genome-edited plants and farm animals and for novel therapies for inherited human diseases. This Special Issue on CRISPR presents different efforts to further improve the efficiency of CRISPR genome editing and provides selected examples of the application of this technology.

Sledzinski et al. provide an overview of different computational tools predicting on- and off-target efficiency of guide RNAs and the pattern of insertions and deletions caused by error-prone repair [1]. They also review programs that quantify the relative amounts of different products of CRISPR genome editing based on the DNA sequencing of a PCR-amplified genomic region around the CRISPR cutting site. Various lesions including CRISPR-induced DSB can occur in the genomic DNA and different repair mechanisms are involved in their removal. Carusillo and Mussolino describe the lesions, the corresponding DNA damage responses, and explain how these repair mechanisms can be used for novel therapies [2]. Off-target cutting of DNA at sites that show similarity to the target site are a major problem in CRISPR genome editing, in particular with respect to gene therapy in humans. Different strategies have been developed to minimize off-target effects, and Naem et al. present the latest developments in that field [3]. To simultaneously quantify both on- and off-target recombination, Sánchez-Hernández et al. develop a novel reporter system that will be useful in identifying conditions that minimize off-target cutting [4].

Following CRISPR-induced DSB, different repair mechanisms compete for closure of the gap. Unfortunately, the error-prone repair described above is much more efficient than the error-free homologous recombination. Is it possible to skew the choice of the repair pathway toward homology-directed repair by small molecules? Bischoff et al. provide insight into low-molecular-weight components that might play this role [5]. For gene therapy, it is important that both the gRNA–Cas9 riboprotein and the DNA repair template reach the target cells at high concentrations. Adenoviral vectors are one of the potential vehicles to achieve this task; Tasca et al. report the current state of art in this field [6]. In addition, Goncalves et al. present novel data showing how third-generation adenoviral vectors can be used to repair human cells with a dystrophin mutation associated with Duchenne muscular dystrophy [7]. If CRISPR genome editing is used on patient-derived tissue specific stem cells or induced pluripotent stem (iPS) cells, only successfully repaired cells should be transplanted back to the patient. To facilitate the selection of such cells, Barbuti et al. present a high-content screening approach to identify repaired iPS cells obtained from a Parkinson patient [8]. The ability of the gRNA–Cas9 riboprotein to bind to a unique site in the genome can be used for the targeted introduction of DSB, and to transport chromatin-modifying enzymes to that genomic site. For this approach, cutting deficient dCas9 proteins is used. Syding et al. show how fusion of dCas9 with enzymes altering epigenetic modifications can be used to treat rare imprinting disorders [9]. In a method called base editing, dCas9 is coupled with enzymes, leading to single-point mutations close to the binding site. Carrington et al. test the efficiency of different base-pair-converting enzymes in germ-line editing of zebrafish [10].

We hope that this Special Issue will provide helpful insight into recent developments in CRISPR genome editing and inspire novel applications of this technique.
